# Anthropogenic Drivers Leading to Population Decline and Genetic Preservation of the Eurasian Griffon Vulture (*Gyps fulvus*)

**DOI:** 10.3390/life11101038

**Published:** 2021-10-01

**Authors:** Monica Pirastru, Paolo Mereu, Laura Manca, Daniela Bebbere, Salvatore Naitana, Giovanni G. Leoni

**Affiliations:** 1 Department of Biomedical Sciences, University of Sassari, Viale San Pietro 43b, 07100 Sassari, Italy; pirastru@uniss.it (M.P.); manca@uniss.it (L.M.); 2 Department of Veterinary Medicine, University of Sassari, Via Vienna 2, 07100 Sassari, Italy; dbebbere@uniss.it (D.B.); snaitana@uniss.it (S.N.); gioleoni@uniss.it (G.G.L.)

**Keywords:** *Gyps fulvus*, genetic integrity, species management, anthropogenic impacts

## Abstract

Human activities are having increasingly devastating effects on the health of marine and terrestrial ecosystems. Studying the adaptive responses of animal species to changes in their habitat can be useful in mitigating this impact. Vultures represent one of the most virtuous examples of adaptation to human-induced environmental changes. Once dependent on wild ungulate populations, these birds have adapted to the epochal change resulting from the birth of agriculture and livestock domestication, maintaining their essential role as ecological scavengers. In this review, we retrace the main splitting events characterising the vultures’ evolution, with particular emphasis on the Eurasian griffon *Gyps fulvus*. We summarise the main ecological and behavioural traits of this species, highlighting its vulnerability to elements introduced into the habitat by humans. We collected the genetic information available to date, underlining their importance for improving the management of this species, as an essential tool to support restocking practices and to protect the genetic integrity of *G. fulvus*. Finally, we examine the difficulties in implementing a coordination system that allows genetic information to be effectively transferred into management programs. Until a linking network is established between scientific research and management practices, the risk of losing important wildlife resources remains high.

## 1. Introduction

Human activities causing alteration of habitats, along with overfishing and hunting, intensive use of pesticide, herbicides and fertiliser in agriculture, the impacts of invasive alien species and climate change are the main causes of biodiversity loss for ecosystems [1,2,3,4]. Based on the latest report on the health of ecosystems, one million of plant and animal species are at risk of extinction because of human activities. Since the rate of species extinction has grown up to hundreds of times higher than in the past 10 million years, many of these species will become extinct within a few decades [5]. However, there are some species that have adapted to these changes and developed new behavioural strategies to survive in close contact with humans, as in the case of vultures. These birds have changed their eating habits by switching their primary food source to farm animals because carcasses of wild animals are scarce and even disappeared in some areas.

Vultures play a key role in maintaining the functioning and health of an ecosystem. As obligate scavengers, they deliver crucial benefits to humans by providing several ecosystem services, such as the regulatory one, which has gained more and more importance, especially in recent times with the spread of intensive breeding [6,7]. Indeed, vultures reduce the rates of transmission of infectious diseases quickly consuming domestic and wild animals’ carcasses. Regarding this role of vultures, the provision of supplementary safe food at artificial feeding stations (SFFS) appears to be the most important management action applied to counter sanitary problems related to intensive breeding and to the conservation of these species sensitive to poisons or drugs present in contaminated carcasses [7,8,9,10,11,12,13,14].

Old World vultures living in Europe, Asia and Africa are members of the Accipitridae family and are closely related to raptors. The New World vultures’ species from America belong to the Cathartidae family, which has been proposed by some authors as being evolutionarily related to Ciconiidae [15,16]. Because of convergent evolution, these two groups of vultures would have adapted their lifestyle to the same ecological niche, developing similar morphology and behaviour [17]. However, more recent studies have disproved the “cathartid-stork hypothesis” and pointed out a Cathartidae sister relationship with Accipitridae [18,19,20,21]. Both Old World and New World vultures are scavenging birds and feed mostly on carcasses of dead animals. Among the morphological and biological characters interpretable as adaptations are bare heads and neck to avoid pollution of feathers when feeding inside carcasses, strong hooked beaks with cutting edges to tear skin apart and feet more appropriate for movement on the ground than to catch prey [16]. A further adaptation is related to the feeding behaviour resulting in complex relationships at both intraspecific and interspecific levels during carcass exploitation [22,23,24,25]. The species of both families developed a static soaring style, perfectly optimised for searching for food over wide areas minimising the energy expenditure. Vultures rely heavily on soaring flight using air thermals [26].

Based on the “IUCN Red list for birds” [27], 11 out of the extant 16 Old World vultures’ species are classified as globally threatened (eight Critically Endangered and three Endangered), while among the seven New World vultures’ species, only two are at risk (one Vulnerable and one Critically Endangered). The causes of the decline of vultures are well known and it is necessary to urgently coordinate and implement the action plan in all its components [28]. In this review, we retrace the evolutionary history of vultures, with particular emphasis on the Eurasian griffon vulture *Gyps fulvus*, and analyse their adaptation to habitat changes, providing useful information for a better management of this species.

## 2. The Genus *Gyps*

The family Accipitridae includes the genus *Gyps* which groups ten Old World vulture’s species. Among them are the extant *G. rueppellii*, *G. africanus* and *G. coprotheres* in Africa; *G. bengalensis*, *G. indicus*, *G. tenuirostris* and *G. himalayensis* in Asia; *G. fulvus* in Europe, Africa and Asia; and the two extinct *G. melitensis* and *G. bochenskii* [29].

The feeding behaviour among *Gyps* vultures is thought to have evolved because of their close association with ungulates, particularly migratory populations in Africa and Asia. Indeed, the temporal and geographic diversification of *Gyps* species coincides with the radiation of Old World ungulates, especially from Bovidae [30,31,32,33]. Such a relationship likely played a significant role in the adaptation and rapid diversification of *Gyps* vultures. In 1983, Houston [34] proposed that their large body size and ability to soar over large distances in search for food are related to the associated migrant distributions and seasonal fluctuations in mortality of ungulates, and that they have consequently become incapable of killing their own prey [26]. However, occasionally, a vulture has been described to kill young and weak individuals without affecting livestock productions [35]. These presumed killing of live and healthy livestock by griffon vultures *Gyps fulvus*, that have no offensive weapons, lacks scientific evidence but has been magnified by the media through the spread of fake news, creating alarm that must be stemmed through careful information campaigns [36,37]. The species of *Gyps* show similar morphological features and can hybridise each other in natural conditions when range overlaps. In central Africa, *G. rueppellii* crossbreeds with *G. africanus*, *G. coprotheres* and *G. fulvus* individuals, the latter migrating in autumn from Spain to Africa, and produces fertile hybrids [38].

## 3. The Eurasian Griffon Vulture: Ecology and Behaviour

Among species of *Gyps*, the Eurasian griffon *G. fulvus* is the most widespread vulture across Europe, Asia and Africa, with a reproductive distribution extending from Kazakhstan and Nepal to southern Europe via the Caucasus [39]. The species is now considered extinct as a breeding species in North Africa, where mainly records of nomadic juveniles or migratory overwintering adults are reported [40]. In Europe, Spain holds more than 95% of the total population and, in global terms, 75% of the world’s griffon vultures [41,42,43]. The remaining European breeding colonies are from the Balkan Peninsula, including Bulgaria, Greece, Macedonia, Serbia and Croatia [44,45,46,47,48,49], along with France, Portugal and the Mediterranean islands of Sardinia, Crete, Naxos and Cyprus [50]. Since this species has an extremely large range, the population trend appears to be increasing, and as the population size is very large, it is currently classified as “Least Concern” according to the IUCN criterion [39].

### 3.1. Habitat

**Figure 1:**
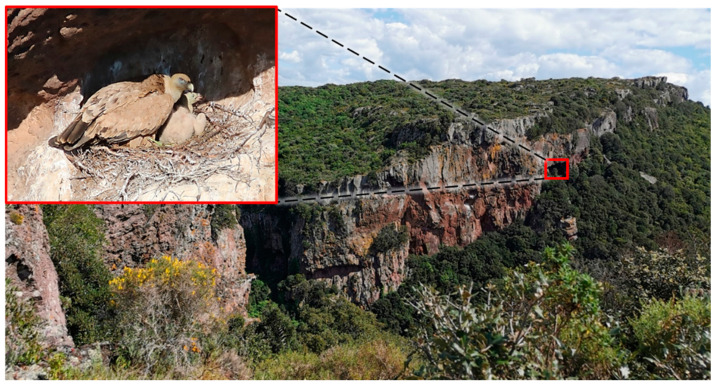
Breeding habitat of the Eurasian griffon with small caves in vertical cliffs. Inset shows an adult attending the chick in the nest (photo: S. Naitana; date: 16 May 2018; location: *Badde Aggiosu*, Bosa—Italy; GPS coordinates: N 40.30163803046105, E 8.516893362179143).

The vertical cliffs are the preferred nesting sites of the Eurasian griffon as well as most species of *Gyps*, except for *G. indicus* and *G. africanus,* both adapted to use trees. The cliffs are high areas of rock with a very steep side, often on a coast, where the erosive action of water generated small caves, ledges and protrusions, making them ideal nesting sites well protected against potential predators [51] (Figure 1). 

In addition, the presence of narrow and high gullies where the air is often forcibly conveyed generating vertical air currents provides important advantages for the take-off and flight [52]. Particularly during the autumn/winter season, these movements of air masses are useful because they compensate for the lack of thermals currents during days with overcast skies. 

**Figure 2:**
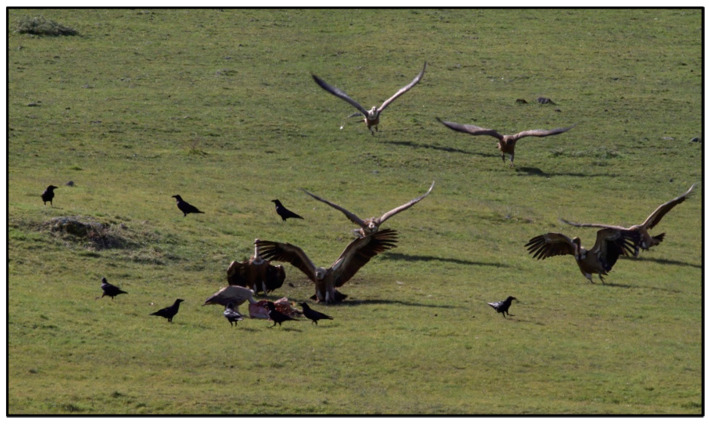
Eurasian griffon surrounding a carcass with crows around waiting for their turn (photo: G. G. Leoni; date: 12 February 2015; location: *Sa Fenalzosa*, Pozzomaggiore—Italy; GPS coordinates: N 40.4184752, E 8.6828675).

Beside the nesting sites, the habitat is characterised by a landscape with large open spaces of scrub and small forests, allowing Eurasian griffon to identify carcasses of wild or domestic animals [53]. The golden eagle (*Aquila chrysaetos*) and the raven (*Corvus corax*) share the same habitat and nest near the griffon vulture’s nests. Both these species represent a potential danger because they often attack or disturb the Eurasian griffon when landing in the nest. Particularly, the raven represents a potential predator of eggs and chicks if left unattended; it also competes with the griffon vulture for carcasses remains (Figure 2).

**Figure 3:**
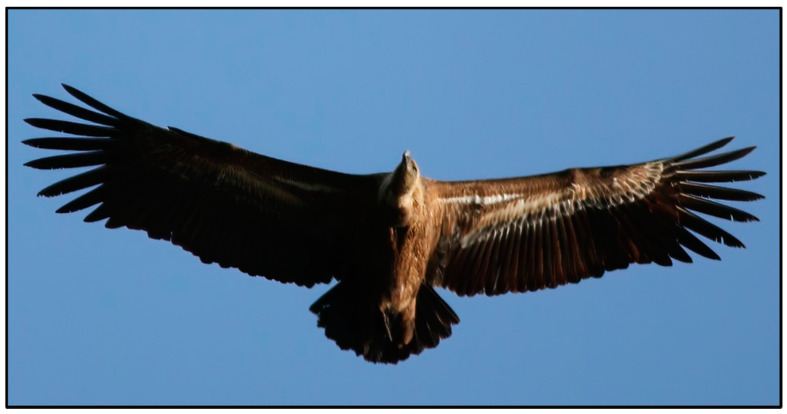
Adult Eurasian griffon in soaring flight (photo: S. Naitana; date: 19 June 2020; location: *Monte Crispu*, Bosa—Italy; GPS coordinates: N 40.333666183095346, E 8.533662984449215).

The Eurasian griffon is sedentary, but it can colonise new areas thanks to the dispersal behaviour of young individuals which are driven to explore new territories by overpopulation or reduction in food availability. The evolutionary adaptation of the wing structure allowed the Eurasian griffon to adopt the soaring flight, an energy-efficient style that minimise the energy cost by using environmental resources such as rising air currents [54] (Figure 3). Due to this flying style, the Eurasian griffon usually flies over land and is very reluctant to cross even short distances over the sea, where the absence of ascending thermal air currents associated to crosswinds may change speed and/or direction of flight [55]. At the beginning of the colonisation process, information derived from habitat features is crucial in determining the colony settlement, while social information between conspecifics becomes predominant when the colony expands [56].

The Eurasian griffon is socially monogamous and shows high nest-site fidelity. Adult philopatry to a general area and site tenacity to a specific nest-site are presumed to gain a benefit in intra-sexual competition for territories due to familiarity with an area [57,58,59,60]. Indeed, the deep knowledge of the breeding site and the consolidated relationships with the conspecifics facilitate individuals in finding food, choosing the best nest-site and taking care of chicks, fundamental aspects for guaranteeing reproductive success. This tendency to prefer the safety of an already known breeding site to the uncertainty of the unknown leads to a reduction in the dispersion rate and favours the formation of colonies, which tend to diversify at a rate proportional to the isolation time, as previously detected in populations on islands [50,61]. Such a behaviour is one of the causes of the site-specific genetic variability highlighted among griffon vulture populations in different geographical areas [50,62].

### 3.2. Food Availability

The extension of the feeding area of the Eurasian griffon is proportional to carcass availability and generally involves a territory of ~25 km in radius in the mainland [63] and of ~9 km in the islands [64], starting from the centre of its nesting site. Every day, it carries out flights such as mopping-up for control and identification of carcasses in the territory, flying much higher if the density of the ungulate population is very low [65]. 

The relationship between the use of feeding sites by avian scavengers and the trophic requirements resulting from the life-cycle phase and individual activities has been pointed out [14,22,66]. Given the considerable parental investment of vultures during their lengthy breeding period [67], they optimise the time spent on searching and obtaining food. In fact, during the incubation and chick-rearing phases, couples attend sites where the food is more predictable and accessible [22].

*Gyps fulvus* has evolved an effective strategy based on rapid exchange of information between conspecifics as a mechanism to counteract the uncertainty deriving from the unpredictability of food. This condition is particularly evident during the period of chick feeding where adults build aerial networks by keeping visual contact each other to cover a wide area. The immobility of the wings in flight contrasts with the incessant lateral oscillation of the head aimed to inspect the territory and locate a carcass [68,69].

*Gyps fulvus* is one of the most sensitive avian species to reductions in food supplies [70]. The drop in the amount of food provided by extensive livestock herds has led to an intensification of the consumption of food by vultures at predictable feeding stations. The link between the food supplied at feeding stations and the increase in antibiotics and in Non-Steroidal Anti-Inflammatory Drugs (NSAID) in plasma and carcasses of the Eurasian griffon vulture has been evidenced [71,72]. The European Medicine Agency (EMA) recognised the risk for *Gyps* vultures from the use of NSAID in animals whose carcasses could be available as food to avian scavengers [73] and several proposals have been carried out to overcome this problem, such as the implementation of control systems, the use of alternative vulture-resistant drugs [28,74,75] and the so-called “One Health Approach” that promotes environmental responsibility and stimulates collaboration between veterinarians, pharmacologists, biologists and ecologists for the health of humans, animals and the environment [76].

To locate food directly, griffon vultures do not use a sense of smell but rely on vision [77]. The griffon has excellent eyesight and in flight can spot an animal carcass from a great distance, and when an individual locates a carcass, lowering its legs, it sends a signal to prepare for landing [78]. The entire carcass is eaten starting from the mouth and anus, in a relatively short time. Observations carried out by Spanish ornithologists have shown how a group of ~30 individuals can identify a carcass in a very short time (2–3 h) and consume a sheep in half an hour [79].

Biotelemetry studies aimed to track movements of sympatric individuals have highlighted how the shared roosts could play as information centre where conspecifics share information about the position of food [80]. The behaviour of conspecifics on foraging success have facilitated the spread of the species [81,82]. As a downside, the efficient information transfer could play an important role in declining populations according to a sigmoidal relationship describing the probability of vultures finding food as a function of vulture density in the habitat [81].

Vultures have one of the most effective immune systems that evolved to protect them from the daily exposition to factors affecting transmission of contagious diseases, such as those deriving from the consumption of the carcasses that produce pathogens and high toxic molecules. The immunity to pathogens present in carcasses is provided from an efficient digestive tract with a low pH value ranging from 1 to 1.5 [83] where, in symbiosis, lives a huge number of bacteria constituting the microbiota producing a bacteriocin with remarkable antimicrobial activity [84]. The presence of the microbiota is the result of the evolutionary ecological strategy for the exploitation of animal carcasses and, consequently, appears to be crucial in conferring protection against pathogens and for survival of the griffon [85]. For these reasons, vultures can also act as a reservoir of pathogenic zoonotic bacteria that can be transmitted to other animal species, increasing their diffusion in wildlife [86,87].

### 3.3. Reproductive Investment

**Figure 4:**
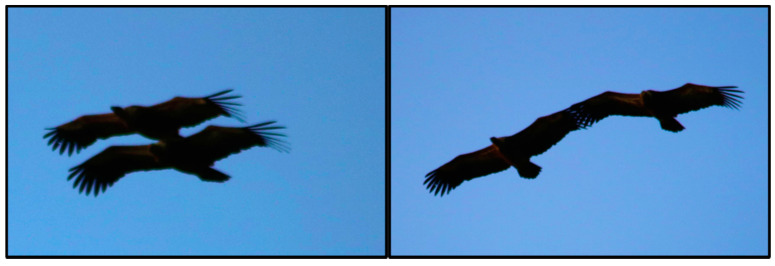
Eurasian griffon performing the nuptial flight (photo: S. Naitana; date: 19 June 2020; location: *Su Caule*, Bosa—Italy; GPS coordinates: N 40.39430966980878, E 8.40493093114848).

*Gyps fulvus* are monogamous birds with a slow lifestyle, producing a single chick that takes a long time to grow and has an increased food requirement [88]. The feeding frequency is primarily regulated by the extent of the foraging range [89]. Since the breeding success depend on the availability of carcasses, a source of food whose presence is unpredictable in space and time, the parental investment is directly related to the carrying capacity of the territory near the nest site [23,40]. The reproductive activity starts with the nuptial flights and the nesting in early winter. Nuptial flights are performed in acrobatic mode by partners who fly one on top of the other or “hand in hand” (Figure 4). 

The latter is a typical flight where two adults fly side by side with the tips of the wings almost touching each other to check the partner’s ability of fly, which is essential in contributing to chick attendance and growth (S.N.—personal observation). During the breeding season, to reduce potential risk of extra-pair copulation in large colony, the Eurasian griffon shows a good average of copulation attempts equal to 71.7, with an average frequency of 1.2 copulations per day [67,88,90]. Different results have been observed in low-density colony [91]. The nuptial flight usually ends on the nest where the partners mate with the cloacal kiss, joining each other and consequently becoming part of the colony. Although it is not a territorial species, during the breeding season, the griffon vulture is inclined to attack intruders to the nest-site. 

The female lays a single white egg 2.5% of its weight, hatching after 52–54 days, with an average productivity ranging from 0.50 to 0.69 of juveniles per breeding pairs per year [63,92] and improving up to 0.82 in a reintroduced colony under favourable conditions [93]. In the early stages of weaning, parents take turns daily in caring for the chick, who is never left alone. Starting from 2 to 3 months, the food frequency is gradually reduced and the chick is often alone in the nest. Most chicks continue relying on their parents and eating in the nest especially during autumn/winter, when food is scarce [88]. 

Juveniles can learn by observation and imitation of adults up to ~5 years after reaching puberty and improve their performances in relation to the ability of flying by using thermal air currents, control and precision when landing and the identification of animal carcasses (S.N.—personal observation). Such a social transmission of information is taxonomically widespread and has already been described in avian species, including some birds of prey [94,95,96,97]. Adults outperform juveniles in challenging thermal soaring conditions and have a greater mastery of soaring static [98]. 

## 4. Eurasian Griffon Population Expansion and Decline 

The availability of animal carcasses and predation residues was fundamental in determining the evolutionary success of the griffon and defining their role as specialised birds in recycling of biomass, in alternative to capturing and killing prey. These birds play a crucial role in keeping ecosystems healthy by contributing to carcass removal, limiting diseases transmission and providing indications of environmental contamination in relation to the quantity of pollutants deposited on their wings [99].

In the past, the main source of food for vultures derived from carcasses of wild animals which, in great numbers, populated most of the habitats overall the world. The Neolithic expansion of human people, the birth of agriculture and animal domestication had a strong impact on the distribution of wild animal populations, changed their natural habitats and caused the extinction of the megafauna. These events led to a continuous reduction in the availability of habitat for wildlife species and therefore, the carcasses of domestic animals such as small ruminants, cattle, pigs and horses became the main source of food for vultures. This phenomenon was particularly evident in Europe, where the vulture drastically changed its habits, switching to a diet almost exclusively based on domestic animal carcasses [12]. A different scenario occurred in Asia and, particularly, in Africa where domesticated cattle was lower in number compared to wild ungulate populations [51,100]. At first, the presence of livestock farms ensured a wide and continuous availability of food, thus creating the conditions for a demographic expansion of the vulture species. However, the dependence on livestock as a primary source of food raised several problems which synergistically led to the decline of vulture populations [101]. Once widespread across the continent, the Eurasian griffon population began to drastically decrease at the beginning of the last century in various European regions such as Italy and France, South-Eastern Europe, the Middle East and throughout the territory of North Africa. The causes of this decline have a single common denominator represented by the expansion of human activities and the emergence of related problems [102]. The alterations are manifold and include fragmentation of the territory for the construction of roads and wind turbines, implementation of new intensive forms in animal breeding, changes in sanitary policies as a result of diseases such as the bovine spongiform encephalopathy, the high diffusion of climbing activity, obsessive photographic hunting, the uncontrolled environmental pollution systems and the disturbing warming of the planet [12].

Among the main negative effects deriving from human activities, hunting, electrocution [103,104,105] and, critically, the use of illegal poisoning baits to control wild and feral predators threatening livestock has been associated with the decline of vultures during 1970–1990 in Europe [106,107,108,109,110,111]. The consumption of a poisoned carcass has a destructive impact on a population, because of the dietary habits of the Eurasian griffon usually builds an aerial information network for the control of the territory and accesses it with a consistent number of individuals (10–30 subjects). This event can cause a vertical drop in consistency in a relatively short time. The effects would be even more devastating if the event occurred during the breeding season, which lasts 9 months of the year. Indeed, the death of the individuals that fed on the poisoned carcass would lead to the disruption of reproductive couples, probably causing an alteration of the male/female sex ratio. In addition, the partner left alone will be forced to leave the nest (resulting in the loss of the egg/chick) to look for a new adult partner and a new nesting site. 

In Spain, the strong sensitivity towards this issue has prompted the regional authorities to set up a structure within the Environmental Supervisory Body, called Unidad Canina Especializada, which inspects livestock farms using specially trained dogs, checking for suspicious substances. The detection of these substances immediately leads to heavy fines. This is a very effective approach because it carries out a strong preventive action in the use of poisonous substances.

More recently, the development of wind farms due to the European objective to increase the proportion of renewable energy increased the rate of disturbance and fatality due to collision of flying vultures with rotating turbine blades [78,112,113,114,115].

It has been revealed that the use of veterinary drugs such as NSAID and antibiotics used in livestock treatments that are toxic to *Gyps* severely affect populations of the Old World vultures [74,76,109,116,117,118,119]. Moreover, the use of poisonous compounds for the fight against stray dogs and against predators of domestic animals such as foxes play a more harmful role [109,110].

In parallel, after the outbreak of bovine spongiform encephalopathy, in 2001, European health policy banned the abandonment of livestock carcasses in the field, and consequently, the availability of food resources declined in some regions by more than 80% [120,121,122,123].

To mitigate the effects of all these negative factors, were firstly created the “vulture restaurants” or Supplementary Feed Stations (SFS) to supply food and help re-establish the decimated populations of these species, and more recently, the light SFS or Supplementary Farm Feed Stations (SFFS) [120,124] which actually represent a fundamental conservation strategy for the management of endangered vultures [125]. These feed stations have been set up away from wind farms and dispersed in the tropic area of vultures where farmers can leave traced carcass to avoid risk of poison and go from unpredictable to predicable food determining a good impact on griffon lifespan [10,78,113,124,126], as well as environmental and economic ecosystem services [127]. The use of SFFS has increased reproductive success and the survival of juveniles in the first year of life, passing from average values of 0.65 and 30% in natural conditions of the colonies [63] to 0.82 and 70% during conservation actions [93], respectively. The conservation actions associated with the establishment of the SFFS may in the future play an effective role in the conservation of the Eurasian griffon as well as the other *Gyps* vulture species. One measure to prevent and eradicate the problem is the establishment of the abovementioned SFFS within individual farms, representing an important conservation tool [22,66].

## 5. Conservation Strategies

The genetic diversity observed among griffon vulture populations could be related to the natal philopatry behaviour expressed by the high nest-site fidelity of the adult. Indeed, although it may lead to reduction in intra-population genetic variability due to increasing in inbreeding levels, natal philopatry is thought to stimulate the rise in genetic variants perfectly adapted to specific ecosystems. Accordingly, natal philopatry expressed for several generations can generate genetic differentiation of local populations, giving rise to a genetic mosaic of the species. Recent studies showed that the Eurasian griffon population living in Sardinia harbours one mitochondrial haplotype never detected in other European populations [61]. Similar results were reported for the griffon vulture population of Crete [50]. These findings suggest that restocking actions could be deleterious and determine negative effects on the genetics of local populations, especially when carried out by means of the introduction of not-genotyped animals. However, restocking is the most effective way to increase the number of individuals and avoid populations reaching the critical level of the minimum viable population size, which is the smaller number of individuals below which a population risks disappearing. This has a greater significance for gregarious species such as *G. fulvus*, in which individuals of a colony help each other to carry out their daily activities. Another important aspect to consider when restocking is carried out is the climate which must be similar between the source region and the area in which the animals will be reintroduced. Indeed, Davidovic et al. [62] pointed out specific morphological differences between the griffon vultures that inhabit the Balkan Peninsula and their counterparts from the Iberian Peninsula. On average, the griffon from the Balkan Peninsula shows a greater body mass and have later hatching time, 1 month after the Mediterranean populations, most likely as an adaptation to a colder climate and delays of herds pasturing due to longer periods of cold. This could explain why only a small percentage of introduced birds from the Iberian Peninsula to Bulgaria survived, suggesting that the continental Balkan population deserves a different conservation strategy, and that reintroduction should not be performed with foreign birds but sourcing from those native populations already present in the Balkan Peninsula.

## 6. The Eurasian Griffon Vulture: Phylogeny and Genetic Diversity

The genetic characterisation of native populations—especially if subjected to pressures causing a significant demographic decline—allow the collection of information useful to preserve local variants that have found to be exclusive to some geographical areas, as in the cases mentioned above of Sardinia and Crete. During the last 20 years, many molecular studies have been carried out aiming to analyse the genetic variability among and within several *G. fulvus* populations from the whole Eurasian continent [50,61,62,128,129,130]. Most of these studies were performed by means of mitochondrial and/or microsatellite markers.

### 6.1. Mitochondrial DNA

Because of its intrinsic characteristics such as maternal inheritance and quick substitution rate, mitochondrial DNA (mtDNA) is the most suitable tool in unravelling evolutionary history and phylogeny of many species [131,132]. If the whole mitogenome sequence is not available, the mtDNA D-loop region can be helpful in estimating the amount of variability and identifying the number of haplogroups and haplotypes in the intra-species level, thanks to its high substitution rate. When some haplotypes/haplogroups are lost or emerged because of events such as migrations, a genetic structure over the geography is generated and geographic patterns of genetic diversity can be detected [133]. Conversely, coding sequences analysis proved to be more suitable for inter-specific investigations and estimates of divergence times between lineages.

In 2005, Lerner and Mindell [134] analysed the phylogenetic relationships within and among the six subfamilies of eagle and Old World vultures by using molecular markers. Overall, 1047 and 1041 base pair of the mitochondrial genes NADH Dehydrogenase subunit 2 (ND2) and cytochrome B (cytB), respectively, along with 1074 bp of the nuclear β-fibrinogen intron 7 (FGB-I7), were sequenced from 55 eagle species representatives of 18 genera, and 13 Old World vulture species representatives of nine genera. The Old World vulture group was found to be polyphyletic with two well-separated subfamilies of different evolutionary origin. The Gypaetinae, which includes four genera different for both genetic and morphological features, split earlier, forming a sister group with Perninae. The remaining vulture species were grouped in the monophyletic Aegypiinae clade that was found closely related to Circaetinae snake eagles.

In 2006, Johnson et al. [128] carried out a phylogenetic study within the genus *Gyps* by analysing the complete sequences of cytB and ND2, plus a fragment of 400 bp length from the mtDNA D-loop from 60 representative specimens of all species. The phylogenetic trees based on the analysis of both single and combined markers sequences supported the monophyly of the genus and its species, whose historical radiation evolved 0.2 to 2.1 million years ago starting from the earliest splitting event that separated *G. bengalensis* from all other *species*. A sister relationship between *G. fulvus-G. rueppellii* and *G. i. indicus-G. i. tenuirostris-G. coprotheres* clades was pointed out, while the *G. f. fulvescens*, before then considered on a morphological basis a subspecies of *G. fulvus*, was found closely related to *G. himalayensis*. 

In a more recent study carried out in 2009 and based on both nuclear (recombination activating gene 1-RAG1) and mitochondrial (cytB gene) sequences, Arshad et al. [129] analysed 260 samples from different localities with the aim to detect any phylogeographic structure among *Gyps* populations. According to what was previously reported [128,134], two main lineages, Aegypiinae and Gypaetinae, were found in the Old World vulture’s evolutionary history. The phylogenetic analysis based on 1026 bp of cytB revealed eight clades of *Gyps* species closely related each other, with *G. bengalensis* early separating about 1.1 million years ago from all other *Gyps,* followed by *G. himalayensis* and *G. africanus*. A sister relationship was furtherly confirmed between *G. fulvus* and *G. rueppellii*, and these two taxa together were a sister group to a clade consisting of *G. indicus*, *G. tenuirostris* and *G. coprotheres*. Similarly to Johnson et al. [128], the most genetically distant species were also found to be those with bordering or even overlapping ranges. For example, lower divergence was found between *G. indicus* and *G. rueppellii* that occupy different continents (Asia and Africa) than between *G. coprotheres* and *G. africanus* in Africa, or *G. tenuirostris* and *G. himalayensis* in South Asia.

**Figure 5:**
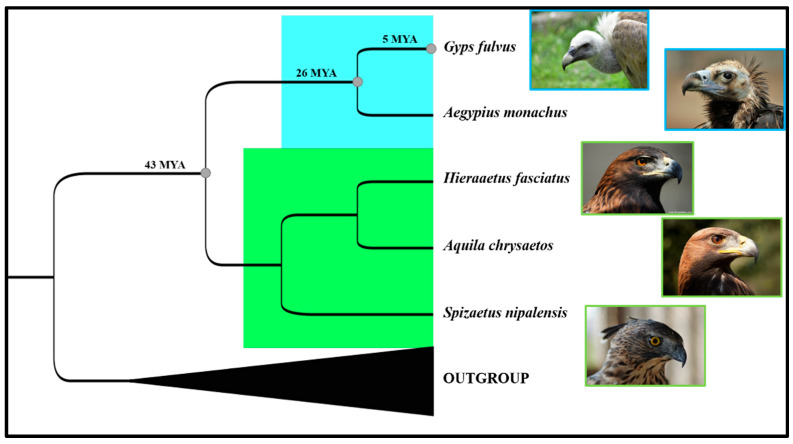
Bayesian tree based on the complete mitogenome sequences, adapted from Mereu et al. 2017, summarizing the phylogenetic relationships between Old World vulture and raptor clades, highlighted in blue and green, respectively. All the nodes retrieved are supported by maximum posterior probability (PP) scores (100). Molecular dating in million years ago (MYA) referring to the nodes marked with full grey circles are reported on the corresponding branch.

An important step was taken in 2017 towards a more comprehensive understanding of the *Gyps* species phylogeny when Mereu et al. [61] sequenced the first entire mitogenome of the griffon vulture. The molecular comparison with other 18 avian mitogenome sequences, including the *Aegypius monachus,* which was the only vulture species to have been characterised when the study was carried out, shed further light on the raptor species evolution. The divergence between eagles and vultures was dated back to about 43 million years ago (MYA), while the birth of the Old World vulture clade and the *Gyps* species’ early radiation occurred about 26 MYA and 5 MYA, respectively (Figure 5). 

Finally, the *Gyps fulvus* species originated about 750,000 years ago. The authors pointed out that the dating of the vulture species appearance overlaps with the diversification of Bovidae [135], something that confirms once again the strict ecological association between vultures and ungulates species. The investigation was carried out on 66 Sardinian griffon samples and identified three mtDNA haplotypes, named Hpt A, Hpt B and Hpt C, with an incidence in the whole sample of 52.9%, 38.2% and 8.9%, respectively. Since the Sardinian colony was subjected to two restocking actions in 1986 (48 individuals) and in 1995 (12 individuals), 22 toe-pad museum specimens collected before 1986 were used to evaluate the impact of these introductions on the mtDNA haplotype frequencies in the native population. Among the museum samples, seven (31.8%) harboured the Hpt A and 15 (68.2%) the Hpt B, while no specimens carrying the Hpt C were found. The authors argue that this change in haplotype frequencies when comparing extant and museum samples could be related to the restocking actions which mostly reintroduced animals with Hpt A, along with a small number of Hpt C individuals. Accordingly, Hpt B could be the most representative haplotype of the pre-decline Sardinian population, whereas the Hpt C was recently introduced sourcing from other populations. 

In a recent study on the mitochondrial D-loop variability of the griffon vulture populations from the Mediterranean islands of Crete, Cyprus and Sardinia, Mereu et al. [50] identified a new haplotype (Hpt D) in the Cretan population. Both in Sardinian and Cretan populations, three haplotypes were detected, two shared (Hpts A and C) and one exclusive to each population: Hpt B in Sardinia and Hpt D in Crete. On the other side, a single haplotype (Hpt A) was found in the Cyprus population. Based on these data, the authors supposed that the higher genetic variability detected in the Cretan and Sardinian populations is the consequence of an evolutionary process affected by long isolation times while the Cypriot colony probably underwent a drastic bottleneck which only the Hpt A survived. The colonisation of these islands would have been characterised by several arrivals of individuals spaced out over time which could have replaced or contributed to enrich the pre-existing gene pool, up to determine the current genetic variability and different expressiveness of the four mtDNA haplotypes among the three populations analysed.

### 6.2. Microsatellite Markers

In 2002, Mira et al. [136] developed five microsatellite markers for the Eurasian vulture *G. fulvus*, providing new molecular tools for population genetic studies and for designing strategies in conservation and reintroduction projects. 

A few years later, the first investigation by means of microsatellite markers was carried out on a network of native and reintroduced Griffon vulture populations successfully restored in Southern Europe, including the native colonies of Israel, Croatia and French Pyrenees (Ossau), one established reintroduced colony in France and four captive founding groups [130,137]. The genetic diversity estimations were similar in all native and reintroduced populations, and overall higher than those measured for other species of vulture in Europe, such as *Gypetus barbatus* [138] and *Neophron percnopterus* [139]. The low F_ST_ levels detected among native populations supported the past existence of high dispersal rates among populations. The native population of Croatia was found to be significantly differentiated from all other populations, probably because of a limited immigration rate into Croatia that, together with small population size, may quickly lead to genetic differentiation. The authors speculated that the present genetic structure is due to the recent isolation of Croatia from other populations caused by the extinction events of intermediate populations between Croatia and Ossau (France) and between Croatia and Israel, which occurred at the end of 19th century and in the 20th century, respectively.

Moreover, high migration rates from Spain into the French colony of Causses were detected, according to asymmetrical gene flow, which over a long period of time could have consequences on local adaptation [140] and deleterious effects on metapopulation viability [141] and should therefore be monitored.

The *G. fulvus* populations from Spain and Israel were found to share their gene pool with populations of *G. africanus*, *G. bengalensis* and *G. indicus* [142] according to some degree of hybridisation, an event that has been reported between *Gyps* species in captivity and under natural circumstances [38]. However, the genetic pattern revealed by Arshad et al. [142] may also be related to homoplasy or retention of shared ancestral states. Overall, the study confirmed what was previously reported [130] regarding the low genetic differentiation among *G. fulvus* populations as a consequence of high mobility and gene flow among them.

A recent molecular study by means of microsatellite markers [62] collected the first genetic data on the Griffon vulture population from Serbia, inhabiting parts of the Balkan Peninsula and representing the last inland population adapted to the continental climate. This griffon population was compared with those from Croatia, Israel (Mediterranean climate) and the Pyrenees in France [130]. Genetic diversity was overall similar to other native populations, including Cyprus and Spain, although the population from Serbia experienced a serious bottleneck during the last decade of the twentieth century. Population structure analysis detected two genetic clusters, one grouping populations from the Balkan Peninsula and the other grouping those from Pyrenees, derived from the Spanish population. The griffon populations from Croatia and Serbia showed higher genetic diversity than those from Pyrenees, with the population of Serbia being genetically most differentiated from all other populations. The Israeli population was found to have admixed ancestry derived equally from the Balkan and the Iberian genetic clusters. Based on this evidence, the authors hypothesised that the Middle East could be recognised as the region from which European populations originated and Israel would be the remnant of the source population from which this species colonised the Mediterranean area. Indeed, it is suggested that during the Last Glacial Maximum (LGM) in Europe the European griffon vulture populations retreated to refugia in North Africa and the Arabian Peninsula. After the end of LGM, Europe was recolonised following two directions, including the way across Gibraltar into the Iberian Peninsula and the way across Bosporus into the Balkan Peninsula. The Israeli population was the only one without a recent bottleneck, a result supporting this hypothesis. Accordingly, it is plausible that during the initial colonisation of Europe from the Middle East, the populations of the Iberian and Balkan Peninsulas went through a founder effect and successive bottleneck, which resulted in the two genetic clusters mentioned above.

## 7. Conclusions

The geographical distribution of the Eurasian griffon appears to be very diversified [39,40,42,44,45,46,47,48,49,50]. In Europe, Spain hosts the largest population, while in other European countries, a sharp population decline has been recorded, native populations have become extinct locally and have consequently been replaced by reintroduced individuals, mostly from Spain, as in the cases of France, Italy, Cyprus and Bulgaria.

Several restocking programs are currently active in Europe with the aim to redistribute the species evenly. Restocking is crucial to contain the numerical decline or repopulate a territory, but if not properly performed, it can determine the dilution, until the definitive disappearance, of local genetic variants. High genetic variation is important for populations to allow the survival of a species under environmental changes. In this regard, emblematic is the case of the griffons of the Balkan peninsula, which, due to their adaptation to the continental climate and the territory where they nest, exhibit a greater body mass and have a hatching time delayed by 1 month compared to the Mediterranean populations. The adaptation to territory and climate also explains why the repopulation of Bulgaria with Spanish griffons did not provide results as expected. Indeed, griffon vulture populations from the Balkan and Iberian Peninsulas are genetically differentiated because of a previously described natal philopatry behaviour. In addition, they probably adapted to different climatic conditions, a not negligible factor in evaluating the potential success of a restocking action. The low success rate observed in Bulgaria after the reintroduction of Spanish griffons suggests that restocking actions should be carried out sourcing from geographically close regions with similar climatic conditions [62].

The native populations, and the genetic and phenotypic variability they show, are the result of a long process of evolution and adaptation to the territory, and therefore represent a historical heritage that must be absolutely protected. Unfortunately, the ecological conditions that have seen the proliferation and spread of *Gyps* species have now disappeared, largely due to the impact of human activities on the environment. However, in some inaccessible and isolated geographical areas, these conditions have been maintained and native populations with genetic variants that have disappeared in other regions have been preserved, as reported for Sardinian and Cretan griffon populations. It is essential that these variants are protected from the risk of being replaced by more common variants harboured by individuals introduced during restocking actions. An example of this danger is what happened in Sardinia, where the mitochondrial haplotype frequencies in the local population significantly changed because of the introduction of Spanish individuals. Indeed, the comparison between museum and extant samples performed by Mereu et al. [61] shows how Hpt B, predominant in the native population, has been largely replaced by Hpt A, which is currently the most frequent in the whole Mediterranean area. The example of the Sardinian population highlights the importance of museum samples to have an estimate of the residual original genetic variability in the current population.

Since genetic data, combined with behavioural, demographic or spatial information, provide a powerful tool for management of wildlife, genetically informed restocking programs started 10 years ago. This preventive control action allows a genetic selection, and, in view of the possible hybridisation phenomena, confirms or not the morphological identification of the specimens, avoiding the risk of introducing unsuitable individuals. This has been the case of a hybrid *G. fulvus*/*G. rueppelli* that was erroneously included in the group of griffons to be released since it was morphologically not distinguishable from pure individuals. This specimen was therefore excluded from the restoking program. 

Despite the abovementioned programs, more efforts can be undertaken in developing partnerships between researchers, institutions and bodies responsible for the management of griffon vulture colonies across Europe. The natural extension of this approach is the further development of programs aimed to not only safeguard the species’ presence but consider the genetic variants that evolved over time in close relationship with the habitats to which they perfectly adapted. For these reasons, it is more important than ever to ensure the transfer of knowledge from the world of scientific research to the structures responsible for the management of the species through the creation of a European network involving all the countries where the species is still present.

As genetic information become progressively available, it is expected that it, along with the massive use of FFS and the awareness of people to minimise the anthropic impact, will play an increasingly important role in the ecology and management of griffons.
